# Sensitivity Comparison of Vapor Trace Detection of Explosives Based on Chemo-Mechanical Sensing with Optical Detection and Capacitive Sensing with Electronic Detection

**DOI:** 10.3390/s140711467

**Published:** 2014-06-27

**Authors:** Drago Strle, Bogdan Štefane, Erik Zupanič, Mario Trifkovič, Marijan Maček, Gregor Jakša, Ivan Kvasič, Igor Muševič

**Affiliations:** 1 Faculty for Electrical Engineering, University of Ljubljana, Tržaška 25, Ljubljana 1000, Slovenia; E-Mails: mario.trifkovic@fe.uni-lj.si (M.T.); marijan.macek@fe.uni-lj.si (M.M.); 2 Faculty for Chemistry and Chemical Technology, University of Ljubljana, Aškerčeva 5, Ljubljana 1000, Slovenia; E-Mails: bogdan.stefane@fkkt.uni-lj.si (B.Š.); gregor.jaksa@ijs.si (G.J.); 3 Institute Jozef Stefan, Jamova 39, Ljubljana 1000, Slovenia; E-Mails: erik.zupanic@ijs.si (E.Z.); ivan.kvasic@ijs.si (I.K.); igor.musevic@ijs.si (I.M.)

**Keywords:** small capacitance measurements, Trinitrotoluene (TNT) vapour trace detection, chemical modification, gas sensor, Micro Electro Mechanical System (MEMS) sensors, vapour trace detection

## Abstract

The article offers a comparison of the sensitivities for vapour trace detection of Trinitrotoluene (TNT) explosives of two different sensor systems: a chemo-mechanical sensor based on chemically modified Atomic Force Microscope (AFM) cantilevers based on Micro Electro Mechanical System (MEMS) technology with optical detection (CMO), and a miniature system based on capacitive detection of chemically functionalized planar capacitors with interdigitated electrodes with a comb-like structure with electronic detection (CE). In both cases (either CMO or CE), the sensor surfaces are chemically functionalized with a layer of APhS (trimethoxyphenylsilane) molecules, which give the strongest sensor response for TNT. The construction and calibration of a vapour generator is also presented. The measurements of the sensor response to TNT are performed under equal conditions for both systems, and the results show that CE system with ultrasensitive electronics is far superior to optical detection using MEMS. Using CMO system, we can detect 300 molecules of TNT in 10^+12^ molecules of N_2_ carrier gas, whereas the CE system can detect three molecules of TNT in 10^+12^ molecules of carrier N_2_.

## 1. Introduction

Detecting vapour traces of explosives and other materials in the atmosphere is a potentially powerful method to reveal the presence of explosive devices and other materials like toxins, *etc*. The principle of the detection method is based on the fact that almost any material, including explosives, emits a rather small, but detectable number of different molecules constituting the target material. Numerous detection techniques have been developed that are capable of detecting explosive devices [1], but their common limitations are rather large size and weight, high power consumption, unreliable detection with false alarms, insufficient sensitivity and/or chemical selectivity, and hyper-sensitivity to mechanical noise associated with a very high price. There is a need for miniature, sensitive and chemically selective sensor systems, typically capable of detecting one target molecule among 10^+12^ molecules of atmosphere in real time.

Chemically surface modified Micro Electro Mechanical System (MEMS) sensors [2–4] are currently the most promising and most popular candidates for the ultrasensitive detection of low concentrations of target molecules in the atmosphere. The detection method is based on optical measurement of the deflection of the sensing micro-cantilever, which is caused by the adsorption of the target molecules on one of the surfaces of the cantilever [5,6]. An alternative to the optical detection method is to measure the change in the capacitance or the resistance of the cantilever [7] or a MEMS device. A very sensitive detection is possible by this method, and Trinitrotoluene (TNT) vapour concentrations in N_2_ of the order of 1:10^+9^ have been reported [8]. However, in all cases, there are several severe problems that limit the application of MEMS in a real environment. The measurement of the cantilever deflection requires a precise optical system, and the detection apparatus is bulky when precision optics with a long optical path has to be used for the precision required. Furthermore, the measurement of cantilever bending is very sensitive to environment influences like vibrations, mechanical shock, and acceleration. Because the cross-section of the sensing cantilever is usually asymmetrical, the cantilevers act as bi-metal devices and are highly sensitive to the temperature changes. This makes chemically functionalized cantilevers very impractical for sensing vapour traces of hazardous chemicals.

An alternative to MEMS is to use Complementary Metal Oxide Semiconductor (CMOS) capacitive sensors with micro-meter-size or sub-micro-meter size electrodes, which were developed recently for various lab-on-chip and bio-sensing applications. Here the surfaces of the capacitor's electrodes are chemically modified in order to enhance the surface adsorption of target molecules, resulting in a slight change of the capacitance of that capacitor. The capacitive method has obvious advantages over the MEMS method, because it is not sensitive to temperature changes and mechanical vibrations, and is also fully compatible with the CMOS production process. While the sensitivity of this method has been reported for lab-on-chip [9] and bio-sensing applications [10], there is no available literature data on the comparison of the sensitivity of comb capacitive sensors with respect to the MEMS optical detection system.

In this study, we report on the sensitivity comparison of two explosive vapour trace detection systems developed by us: **C**hemo **M**echanical sensor with **O**ptical detection (CMO) and **C**omb **C**apacitive sensors with **E**lectronic detection (CE). Both systems were tested for TNT and Hexogen (RDX) explosive sensitivity in the N_2_ carrier gas and in the air. We found that the sensitivity of our CMO system is in the range of approximately 300 molecules of TNT in 10^+12^ molecules of N_2_ gas, while the sensitivity of our CE system is of the order of 3 molecules of TNT in 10^+12^ molecules of carrier gas N_2_. The sensitivity of the CE system is more than two orders of magnitude better than the sensitivity of the CMO system. It should be noted that the degree of the electronic integration of our CE system is much higher than the integration of our CMO system, which is built with discrete electronics. Even if we try to integrate the electronics for the CMO system, it is very difficult to integrate the laser and to reduce the optical path without losing further sensitivity.

The article is organized as follows: Section 2 describes the principles of the operation of the chemo-mechanical sensor with optical detection, the structure of the cantilever with chemical modification of the sensing surface, the optical detection principle, and an estimation of the Signal to Noise (*S/N*) ratio supported by our measurements. Section 3 examines the capacitive sensor with electronic detection, and describes the principle of operation, the capacitive sensor design, the fabrication and modification, the architecture of the electronic detection system together with the estimates of achievable *S/N* ratio or detection sensitivity, the implementation of a complete demonstrator, and our measurements that support the detection sensitivity estimates. Section 4 describes vapour generator, used in testing the sensors' response, while Section 5 presents the sensitivity comparison of both detection systems. Section 6 concludes the article.

## 2. Chemo-Mechanical Sensing with Optical Detection

### 2.1. Principles of Operation

The invention of the Atomic Force Microscope (AFM) [11] has triggered great interest in using micromechanical devices (MEMS) for quick and reliable detection of small concentrations of target molecules in air and solutions. AFM cantilevers are primarily used for surface imaging, where an atomically sharp tip senses the tip-surface interaction at the sub-nano-newton level. Besides surface imaging, the AFM cantilevers are, in principle, very sensitive force sensors that are able to detect forces in the 10^−12^ Newton (pN) range, and we can measure the cantilever deflections below nm. This makes it possible to measure single molecular forces, and the forces due to the molecules adsorbed on functionalized surface of the cantilever [12].

**Figure 1.**
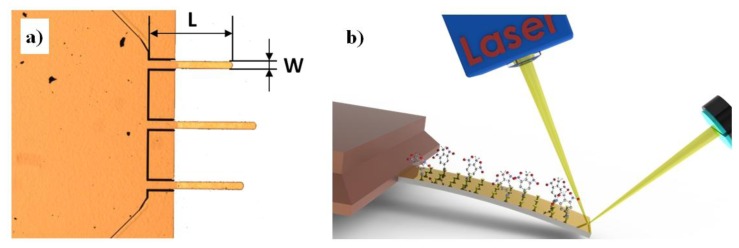
(**a**) Tipples Si rectangular Atomic Force Microscope (AFM) cantilevers of different length (*L*), width (*W*), and thickness (*T*), with force constants ranging from 0.03 to 1.75 N/m, are used as a base for sensitive detection. (**b**) The principle of chemo-mechanical sensing of vapour trace detection uses a focused laser beam and a position sensitive photodiode for optical readout of bending of a chemically functionalized AFM cantilever.

For this purpose, commercially available, micro fabricated silicon cantilevers, which are typically 100–350 μm long, 20–25 μm wide, and 0.5–1 μm thick (Figure 1a) and with force constants ranging from 0.03 to 1.75 N/m, are functionalized on one of the two sides with a thin molecular layer that shows an affinity to the target molecules present in the environment. The surface coverage of the functionalized surface by target molecules is increased in the continuous process of surface adsorption and desorption, and generates either compressive or tensile surface stress which eventually results in a bending of the cantilever. This small bending can be measured either optically or electrically. In optical detection, a focused laser beam is reflected from the cantilever and the cantilever bending causes a deflection of the reflected beam [12]. This deflection is measured very precisely using a quadrant photodiode detection scheme (Figure 1b), a principle invented for the Atomic Force Microscope (AFM) microscope [11]. Another possibility is to measure a change in the resonant frequency of the cantilever due to the mass loading [13,14].

The detection sensitivity of chemically functionalized MEMS is in the range of 1:10^+9^, which makes MEMS devices extremely interesting for the realization of the electronic nose. However, it is difficult to miniaturize their optical detection system, as they inherently need a large optical path to obtain such high deflection sensitivity. MEMS are also extremely susceptible to the environment's mechanical noise and very sensitive to temperature changes because of the thin layer of an optically reflective metal, applied to one side of the cantilever, which therefore forms a bi-metal. In our cantilevers, a thin layer of Au was deposited on one side, which was used as a binding surface for receptor molecules, using gold-thiol-chemistry. Later, to avoid very high sensitivity of the cantilevers to the temperature changes and thus complicated and time consuming temperature stabilization procedures, we developed and used a technique to chemically functionalize the surface of a silicon cantilever directly, without the need of a metal coating.

### 2.2. Chemical Functionalization

**Figure 2.**
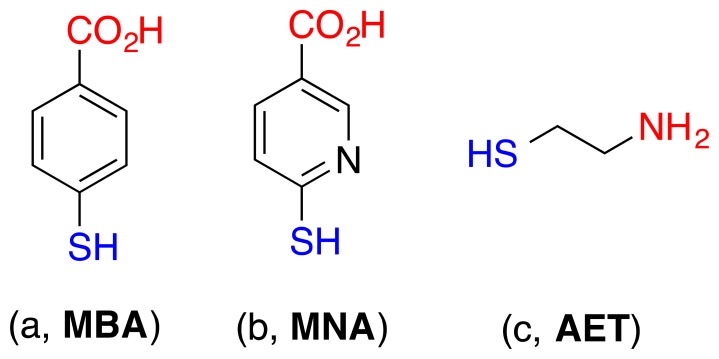
Chemical modification of gold-coated cantilever surface with: (**a**) 4-mercaptobenzoic acid (MBA), (**b**) 6-mercaptonicotinic acid (MNA), and (**c**) 2-aminoethanethiol (AET).

The surface modification of cantilevers was performed according to standard procedures [15]. The cantilevers were cleaned in acetone and then in ethanol, followed by a deionized water wash prior to the modification. A degased ethanol solution of 4-mercaptobenzoic acid, 6-mercaptonicotinic acid, or 2-aminoethanethiol (6 × 10^−3^) (see Figure 2) were prepared in glass vessels, respectively, which were previously cleaned with piranha solution (3:1 H_2_SO_4_/H_2_O_2_) (**Caution**! *Piranha solution can react violently with organic compounds and should be handled with care*). The clean, gold-coated cantilevers were immersed in the thiol solutions for 24 h at 25 °C. After the completion of the modification, the cantilevers were rinsed with absolute ethanol and blown dry with argon before use.

**Figure 3.**
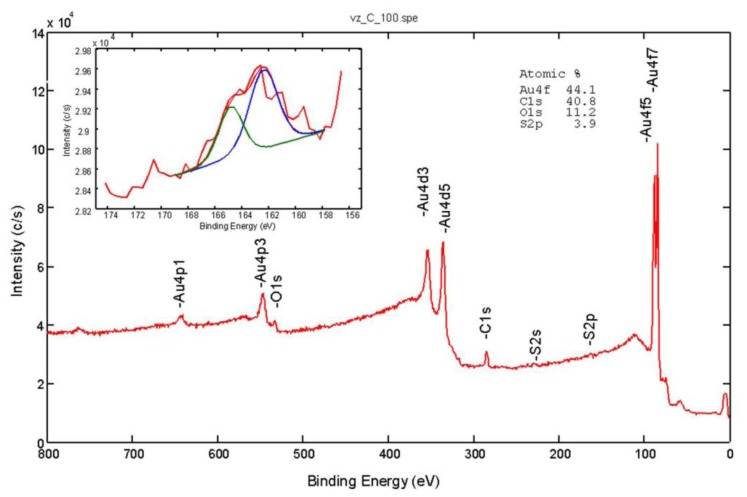
The representative X-Ray Photoelectron Spectroscopy (XPS) spectra analysis of the surfaces of the cantilevers modified with 4-mercaptobenzoic acid (MBA).

The X-Ray Photoelectron Spectroscopy (XPS) or Electron Spectroscopy for Chemical Analysis (ESCA) analyses were carried out on the PHI-TFA XPS spectrometer produced by Physical Electronics, Inc. (Chanhassen, MN, USA). The analysed area was 0.4 mm in diameter and the analysed depth was about 3–5 nm. Sample surfaces were excited by X-ray radiation from a monochromatic Al source. The surface composition was quantified from XPS peak intensities considering the relative sensitivity factors provided by the instrument manufacturer. Two different locations were analysed on each sample, and the data were averaged. The surface composition and chemical bonding of the modified surfaces were characterized by the XPS technique. Figure 3 shows typical survey XPS spectra from the Au surface modified with 4-mercaptobenzoic acid. The peaks of carbon, oxygen, sulphur, and gold were identified in the acquired XPS spectra.

In accordance with the molecular structures of 4-mercaptobenzoic acid, the C and O peaks increased and the Au peak decreased compared to the unmodified Au surface; in addition S peaks appeared after the modification. These results confirmed successful bonding to the Au surface. The inserted spectrum in Figure 3 is high-energy-resolution S 2p XPS spectrum. As expected for the spin-orbit splitting of the S 2p signal we identified a doublet (2p_3/2_ and 2p_1/2_) with an intensity ratio 2:1, respectively. The main S 2p_3/2_ signal at 162.4 eV is in the energy range expected for thiolate bonded to the gold surface.

### 2.3. Optical Detection System

**Figure 4.**
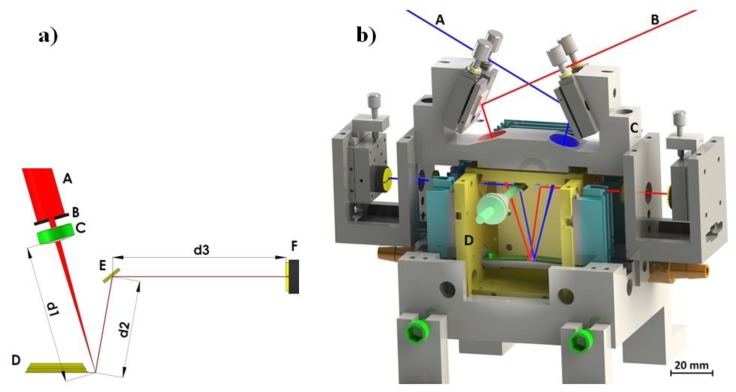
Optical detection principle and implementation. (**a**) A collimated diode laser light (λ = 635 nm, 5 mW) (A) passes through small aperture (B) and is focused at the backside of a cantilever (D) using *f* = 50 mm spherical lens (C). The reflected beam is directed onto a position sensitive photodiode (F) using a rotatable mirror (E). Distances d1, d2, and d3 are 48.3 mm, 34 mm, and 60.3 mm, respectively. (**b**) The optical detection system used for a precise detection of a functionalized (blue beam—A) and a reference (red beam—B) cantilever bending. The outer (gray—C) parts of the system are made of stainless-steel and designed to be as rigid as possible. The inner (yellow—D) temperature-controlled chamber is made of gold-plated copper, and thermally stabilized by several Peltier elements with an accuracy of a few mK.

The deflection of a cantilever sensor can be detected by various techniques [16]. The most frequently used method is beam deflection readout by the “beam bounce method” [17]. The method is relatively simple and is used to obtain cantilever deflection with very high accuracy (a schematic representation of our system is given in Figure 4a). A collimated light from a 5 mW laser diode with wavelength λ = 635 nm passes through a small aperture (diameter around 1 mm) and is focused on the backside of a cantilever using *f* = 50 mm spherical lens. The reflected beam is directed onto a position sensitive four-quadrant photodiode (PSD) using an adjustable mirror. The PSD consists of four closely spaced segments, and the photocurrent from each segment is proportional to the amount of light impinging on that segment from the cantilever. Thus, the changes in position of the reflected light can be precisely determined by measuring the difference in photocurrents from each photodiode segment using a differential amplifier. Cantilever displacements of the order of 0.1 nm are easily detected in this way. Another optical method for precisely detecting cantilever bending is the optical interferometric method [16]. It is much more sensitive, but requires an elaborate optical set-up.

The electrical methods of detection of cantilever bending include the Scanning Tunnelling Microscope principle of operation. A sharp conductive tip is positioned in close proximity to the back side of the AFM conducting cantilever and the tunnelling current between the tip and the cantilever is measured. This method was used in the first AFM experiment by Binnig and Quate [11]. In comparison to the beam deflection system it is much more sensitive, but is also quite complicated to set up and relatively bulky and thus rarely used. Other electrical methods of detection of cantilever bending include piezoresistive and capacitive detection [18,19]. While the piezo resistive method is quite handy, the force sensitivity is nearly two orders of magnitude lower compared to the optical detection [20,21] and thus impractical for high sensitivity sensor devices.

The complete optical detection system is shown in Figure 4b. The outer part of the system is made out of stainless steel and serves as a carrier for mirrors, lenses, PSDs, and cantilever holders. This part has to be as rigid as possible to minimize any vibration between components of the measuring system. It is mounted on a vibration-isolated optical table. The inner part is made out of polished and gold plated copper and serves as a temperature stabilization cage. Its temperature is stabilized by 12 Peltier elements to a temperature within 1 mK. The copper cage is mounted onto a stainless steel outer part by only four Teflon holders in order to minimize the heat transfer between the two parts. All light beams enter and exit the inner part through thin glass windows. The testing gas enters the system from one side and exits the system on the other.

The system consists of two mirror layouts for beam deflection readout and uses two different cantilevers for the operation (red and blue paths in Figure 4b). Simultaneous and independent measurement of the deflection of both cantilevers is possible. The top mirrors are mounted using tilt mirror mounts and used to direct the beams from the laser diodes onto the cantilever ends. The laser light enters the system through a small aperture and is focused using a spherical lens with a focal length of *f* = 50 mm. The reflected light is directed out of the inner copper part by mirrors, mounted on rotatable holders. The exact position of the reflected beam relative to the PSD is adjusted by translation mounts which hold the PSD. Each of the two cantilevers is mounted on a holder with a flat spring, and the distance between them in the system is about 1 mm. Next to the cantilevers is a miniature Pt100 Resistance Temperature Detector (RTD) sensor, which measures the temperature of the atmosphere in the vicinity of both cantilevers with milli-Kelvin accuracy. The testing gas enters the system on one side and exits on the other.

### 2.4. Measurements of the Cantilever Response to TNT Vapours

The cantilevers with standard Au reflective coating surface modified according to Section 2.2. showed huge response even to pure N_2_, which was due the temperature induced bending. Even small variations of the temperature of the incoming gas caused a notable PSD signal. It was shown a long time ago that standard Au-coated AFM cantilevers, such as STMS-06AU (Park Scientific, Sunnyvale, CA, USA), are very sensitive thermometers and bend for ∼0.2° for 1 K temperature difference [21]. The temperature sensitivity of the z-deflection of the cantilever tip is estimated to ∼0.1 nm/1 mK, which is indeed the order of magnitude, observed in our experiments. Because of such high temperature sensitivity, Au-back-coated AFM cantilevers were abandoned in further experiments and cantilevers without any coating were used. The best results were acquired by using uMasch CSC12 tipless Si cantilevers (Innovative Solutions Ltd., Sofia, Bulgaria) with no coatings on either side of the cantilever. After being chemically functionalized (as described in Section 3.2.2.) the bottom side of the cantilevers was ion sputtered in an ultra-high vacuum with Ar^+^ ions (15 min at 500 eV ion energy and ion current of 1 μA/cm^2^). The sputtering process destroys and partially removes the active molecular layer, as checked by the XPS analysis of clean, functionalized, and sputtered surfaces.

All measurements were performed using a homemade vapour generator described in Section 4.1. This vapour generator shows excellent temperature stability of output gas, good control of gas flows and mixture ratios, and is vital for reliable measurements. Prior to measurement, the vapour generator and the measuring system were extensively purged with clean nitrogen (4.6 quality) for several hours. The concentration of targeted molecules in the carrier gas was regularly checked by using a cold trap and doing NMR and gravimetric analysis.

**Figure 5.**
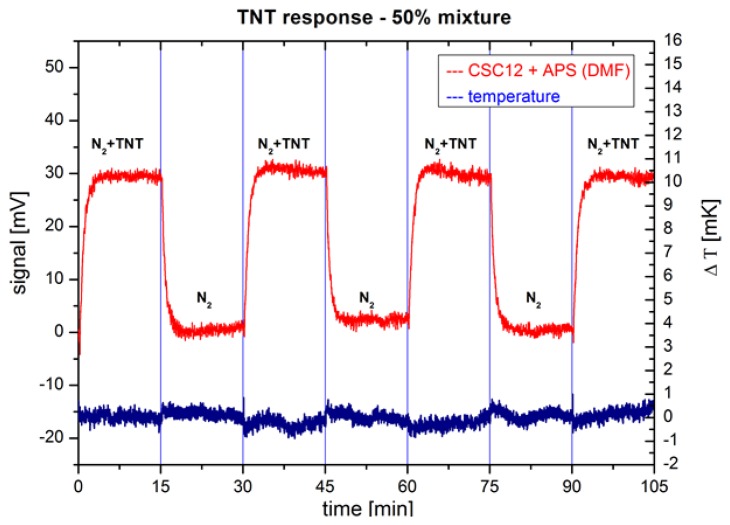
Measured responses of CSC12 cantilevers functionalized with APS molecules (red) on switching between pure N_2_ and mixture of N_2_ and 50% vapour pressure of TNT at room temperature. Temperature changes in the system (blue) due to switching between the two gases are below 1 mK.

Figure 5 shows the response of a CSC12 cantilever with *k* = 0.03 N/m and functionalized with 3-trimethoxysilyl-propan-1-amine (APS) molecules on switching between a clean N_2_ gas and a 50% saturated vapour pressure of TNT at room temperature. The maximum response from PSD of approximately 30 mV is achieved in about three minutes. The cantilever shows stable responses on TNT molecules over several hours of operation. In the same figure the temperature measured close to the cantilevers is plotted. The temperature is stable to about 1 mK during the time of the experiment and shows no change during switching between the two gases.

**Figure 6.**
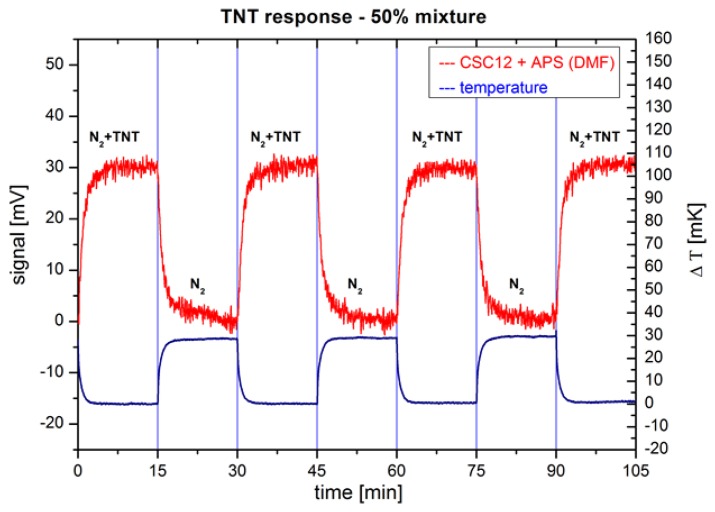
Measured responses of CSC12 cantilevers functionalized with APS molecules (red) on switching between pure N_2_ and a mixture of N_2_ and 50% vapour pressure of TNT at room temperature. In this measurement, the temperature changes in the system (blue) due to switching between the two gases are deliberately set to approximately 30 mK.

To verify that the cantilever bending is indeed the consequence of the surface adsorption of target molecules with the APS active layer and not due to a bimetal effect or some other temperature-related change in the measuring system, we changed the temperature of the clean N_2_ gas by a few tens of mK. Figure 6 shows the response of the same cantilever on switching between a clean N_2_ gas and a 50% saturated vapour pressure of TNT. The measured temperature changes of the clean N_2_ gas are of the order of 30 mK, while the functionalized cantilever response remains almost unchanged at about 32 mV.

The sensitivity of the chemo-mechanical sensing with optical detection can be estimated from the measured results (Figure 6) using Equation (1):

(1) $$S_{\text{TNT},\text{CMO}}≅\frac{0.5\cdot X_{\text{targ}}\cdot \sigma_{\text{CMO}}\cdot 3}{\Delta N_{\text{TNT},\text{CMO}}\sqrt{BW_{\text{CMO}}}}=\frac{3\cdot 10^{-10}}{\sqrt{\text{Hz}}}$$

where the parameters are taken from the results of the measurements presented in Figure 6. They are: *X*_targ_ = 10^−9^ is the estimated density of TNT molecules in the carrier gas at vapour pressure and room temperature, σ_CMO_ = 2 mV is a standard deviation of the readings after the response is stabilized, ΔNTNT,CMD = 30 mV is the step size of the average response and BW_CMO_ = 0.1 Hz is the Nyquist bandwidth of the reading. The acronym CMO stands for Chemo-Mechanical sensor with Optical detection.

## 3. Capacitive Sensing with Electronic Detection

### 3.1. Principle of Operation

**Figure 7.**
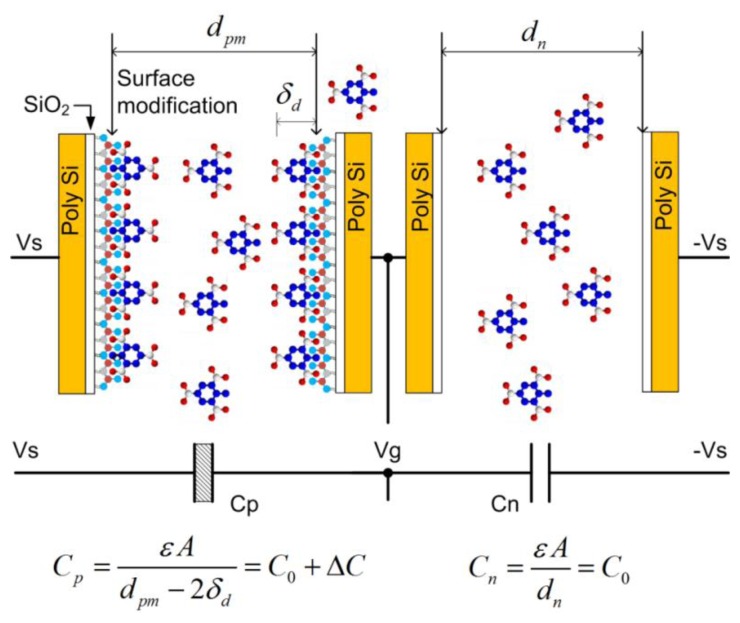
Principle of vapour traces detection of target molecules using a pair of differently functionalised comb capacitors. The chemical asymmetry of the capacitors' electrodes results in the asymmetry of the capacitance due to the preferential adsorption of target molecules on functionalized electrodes.

The essential part of the electronic detection measurement system is a modified differential capacitive sensor, shown in Figure 7. It is composed of two planar capacitors with inter-digitated electrodes with comb-like structure (COMB), covered with a thin layer of silicon dioxide: *C_p_* is chemically modified, while *C_n_* is not. Both capacitors are near each other, so on average, an equal number of target molecules is present in the atmosphere above capacitors. As one of the sensors is chemically modified with a molecular monolayer enhancing the surface adsorption of target molecules when compared to the non-functionalized capacitor, the average surface coverage of targeted molecules is increased on the modified capacitor.

The time-average capacitance of the capacitor *C_p_* (Figure 7) is increased because of adsorbed molecules to the functionalized plates of that capacitor; the plates are now partially covered with an extra monolayer of targeted molecules, which have a dielectric constant larger than that of the air. The capacitances of the two capacitors in a differential pair are now slightly different, which can in principle be measured using appropriate low noise electronics. A rather straightforward calculation using Equations (2) and (3) shows that the detection of small number of adsorbed target molecules is possible if the measurements are performed with extremely low noise electronics.

### 3.2. Capacitive Sensor Design and Fabrication

#### 3.2.1. Comb Sensor

**Figure 8.**
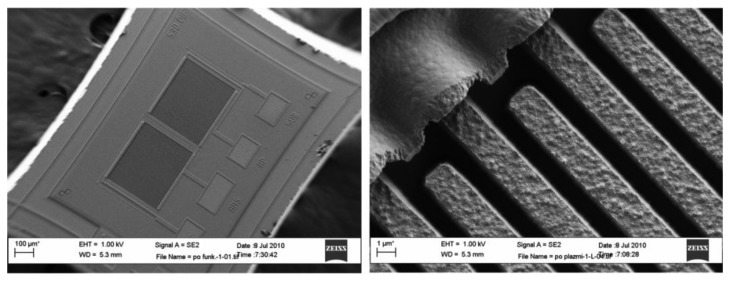
Scanning electron microscope (SEM) micrographs of a comb differential sensor. A pair of capacitors, forming the differential sensor is shown on the left and the detail of one comb capacitor is shown on the right.

Figure 8 shows the scanning electron microscope (SEM) micrograph of an implemented comb sensor using a modified 1 μm MEMS process. Poly silicon comb fingers are 1 μm apart and 2.5 μm high; they are posted onto a thick layer of silicon dioxide and covered with approximately 10 nm of silicon dioxide. One capacitor consists of 51 fingers with length of approximately 350 μm. They are interconnected using Al metal lines forming a capacitor with approximately 0.5 pF capacitance. In the process following MEMS fabrications, both capacitors of each differential pair are first chemically functionalized with TNT receptor molecules (see Subsection 3.2.2). The functionalization layer on one capacitor is later removed using selective laser erosion (see Subsection 3.2.3) to obtain two chemically different capacitors. After processing, the capacitors do not have exactly the same capacitance. The 3σ statistical variation of the matching accuracy can reach up to ±10%; therefore, the initial difference might be as big as 50 fF. The difference is reduced during automatic calibration procedure at the beginning of the measurement cycle (see Subsection 3.3.3.).

#### 3.2.2. Surface Functionalization by Silane Sensing Monolayers

**Figure 9.**
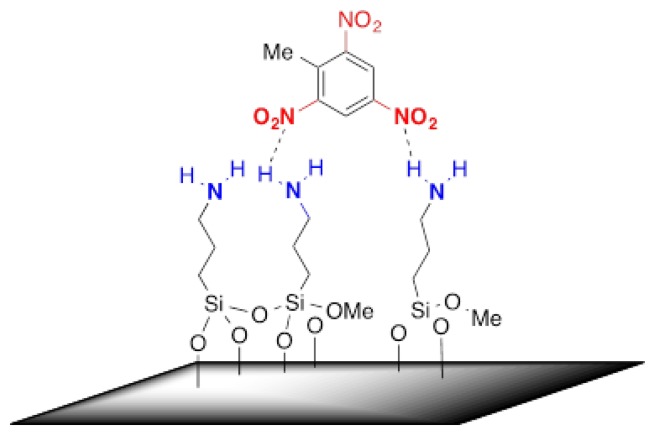
Schematic diagram, representing the surface modified with 3-aminopropyltrimethoxysilane and its sensitive binding mechanism to TNT molecules.

In our experiments, we selected a surface modification using 3-aminopropyltrimethoxysilane to recognise –NO_2_ containing explosives trough the hydrogen bond interactions between the NH_2_ and –NO_2_ functional groups [22], as shown in Figure 9. Moreover, the partial charged complex pairs are not excluded, resulting in enhanced sensitivities in the sensing of TNT molecules [23]. Furthermore, the modified surface also provides a unique stereo selectivity to the meta-nitroaromatics.

**Figure 10.**
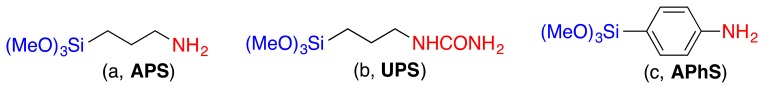
Chemical modification of SiO_2_ surface with: (**a**) 3-trimethoxysilyl-propan-1-amine (APS); (**b**) 3-triethoxysilylpropylurea (UPS); and (**c**) trimethoxyphenylsilane (APhS).

**Figure 11.**
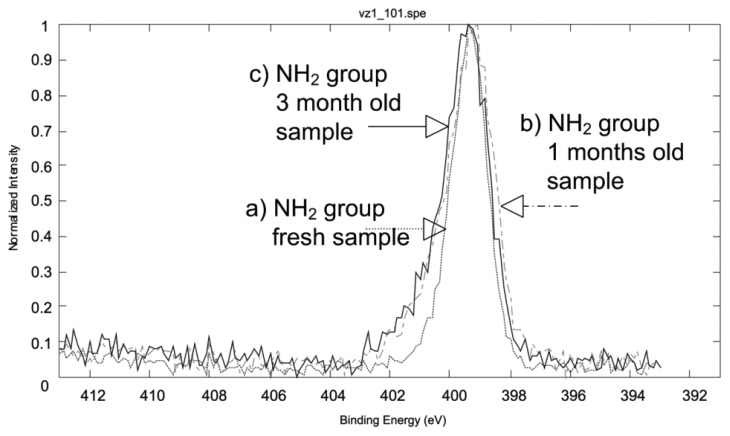
The result of XPS analysis of the APS modified SiO_2_ surface: (**a**) XPS of –NH_2_ group of a freshly prepared sample; (**b**) XPS of a –NH_2_ group of 1 month old sample; and (**c**) XPS of a –NH_2_ group of three months old sample.

The surface, used for molecular sensing, is a SiO_2_ surface covered with layer of coxyalkylamino and trialcoxyarylamino silanes. Generally, the modification of the surface was carried out by dipping the sensors into a diluted solution of the corresponding silane in the organic solvent for an appropriate period of time at room temperature. It was found that different procedures had to be applied for successful modification, with regards to the silane used (Figure 10). Depending on the silane, solvents with different polarity were used to assure its solubility. For the modification using APS organosilane (3-(trimethoxysilyl-propan-1-amine)), a 10 mL of 3 mM dimethylformamide solution of the corresponding silane was prepared in dry flat-bottomed glassware under argon atmosphere. The SiO_2_ surface of the substrates was first treated with oxygen plasma, and then the substrates were immersed in the corresponding solution. The modification process was carried out for a period of 6 h at room temperature. Upon completion, the modified SiO_2_ COMB sensors were removed from the solution and rinsed several times with dimethyl-formamide and methanol to remove any organic residue. Finally, the sensors were thoroughly dried under an argon stream. The surface modification of the sensors with APhS (trimethoxyphenylsilane) and UPS organosilanes (1-3-triethoxysilylpropylurea) was identical; the only difference was the use of dry toluene as a solvent (Figure 10). The XPS measurements confirmed the presence of an amino group on the SiO_2_ COMB sensor surfaces for those conforming to successful modification with the listed organosilanes (see Figure 11), which shows XPS results for a freshly prepared sample, a one month-old sample, and a three month-old sample. The analysis results prove the stability of our modification process.

#### 3.2.3. Surface de-Functionalization by Laser Erosion

Since both comb capacitors of one sensor are covered with a monolayer of sensing molecules during the functionalization process with silanes, one of the sensors has to be de-functionalized in order to obtain a differential response. The removal of surface-adsorbed silane molecules has to be localized and well controlled, as the surface of the active functionalized area is only 250 by 350 μm, and the separation between two capacitors that form a differential pair is only 30 μm. We developed a laser-assisted process of removing the silane monolayer based on controlled surface illumination with focused Ar^+^ laser light with a wavelength of 514.5 nm. The surface of the sensor was positioned in the focal plane of a 60× objective, focusing the 750 mW laser light to a diffraction-limited spot of ∼1 μm diameter. During a 10 s illumination, the local temperature of the sensor surface increased to approximately 80–100 °C. The sensor was moved in a regular manner, so that the illumination spot gradually scanned the whole silane functionalized surface of the selected comb capacitor. The effects of laser-assisted erosion were clearly observed on the test surfaces by the XPS. The results indicate that a five second illumination with a fully focused power of 750 mW is sufficient to decompose and remove different silane modification monolayers on the SiO_2_ surface of silicon.

### 3.3. Electronic Detection System

#### 3.3.1. The Architecture

**Figure 12.**
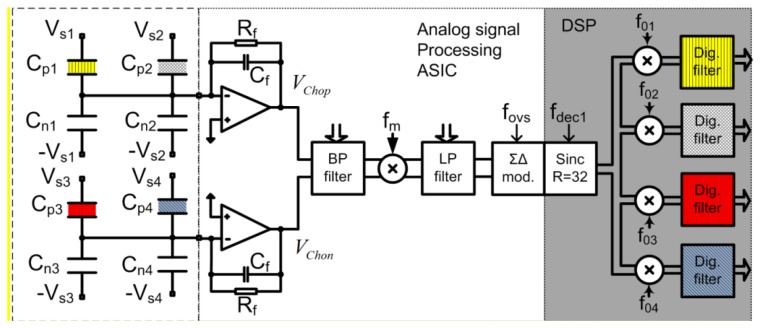
Signal processing block diagram of the low noise electronic measurement system that can process signals from four differently modified sensors.

The detailed architecture, principle of operation, calculation of Signal to Noise (*S/N*) ratio and system level aspects of the electronic detection systems are completely presented in the Supplementary information file. The electronic detection system is based on extremely low noise integrated modified lock-in amplifier architecture and circuit, which is optimised for the array of sensors, and which is able to process signals from four differential sensors that are generally differently functionalised. Each sensor is driven with excitation signal having different frequency, and thus the response from the whole array could be extracted in parallel in “real time”. Figure 12 shows a simplified block diagram of a signal processing electronics implemented in 0.25 μm Bipolar CMOS Double diffused Metal Oxide Semiconductor (BCD) process used to extract information from four sensors. It is essential that the electronics and the sensors are carefully optimised to each other because only in that way it is possible to reach the extreme detection level.

The signals at the outputs of both charge amplifiers *V*_chop_ and *V*_chon_ include information on the capacitance differences of all the sensors connected to the particular amplifier; each sensor response appears at a different frequency. The complete signal is amplified using a fully-differential and programmable low-noise band-pass amplifier. The resultant signal level is well above the thermal noise level of the following blocks. The composed and amplified signal is mixed with a square-wave signal with frequency *f*_m_ in a precision passive mixer [24,25]. The amplitude of the corresponding spectral component is proportional to the excitation signal amplitude (assumed to be constant during the measurements) and corresponding capacitance difference of the sensor, which is caused by the adsorbed target molecules onto the modified capacitor. The signal levels of the spectral components carrying the information are well above the quantization and thermal noise level of the Analog to Digital Converter (ADC), while remaining higher harmonics fall outside the band of interest and are attenuated with a digital decimation filter implemented in the DSP. Further signal processing is digital and consists of digital mixers and LP digital filters. Digital mixers translate the signals from *f_ox_* (*x* = 1,2, …,4) down to DC in a four separate digital channels. The remaining spectral components are further attenuated in digital low-pass filters. The DSP output rate is approximately 14 samples per second with the word-length of 32 bits. The results are transferred to the PC via USB interface, where further signal processing (averaging), storage and presentation are taking place.

#### 3.3.2. Generation of Excitation Sensor Signals

Excitation sensor signals are generated in the Field Programmable Gate Array (FPGA) and transferred to the Application Specific Integrated Circuit (ASIC), where further shaping and amplification takes place. The amplitudes and the DC levels of each excitation signal can be adjusted. They are connected to the corresponding sensor's capacitors and provide the possibility of transferring the information from slowly varying sensor capacitances to the trans-impedance charge amplifier at a high frequency, well above the flicker noise corner frequency. The whole measurement system looks like a modified lock-in amplifier [26,27], using double-mixing architecture to sense and amplify at high frequency above the 1/*f* noise corner of the CMOS charge amplifier. Each spectral component is not immediately reduced to the DC because, in that case, the 1/*f* noise and the offset voltage of the ADC would influence the results. The amplitudes, DC levels, and frequencies of excitation signals are programmable, and we can adapt each excitation generator to a particular sensor. At the moment up to four differential sensors are connected to one measurement channel according to Figure 12. Each sensor is driven with a square-wave excitation signal with slightly different frequency *f_sx_*, where all the signals are coherent [28]. One measurement channel can process the signals of four differential sensors. The “DC” signal-processing happens only in the DSP, and thus all DC drifts and 1/*f* noise problems of the analogue electronic modules are removed. As a result, the noise properties are improved.

#### 3.3.3. Calibration

Matching properties of the sensors and additional modification monolayer on one capacitor may cause the initial capacitance differences of up to 10%, which would drive the measurement channel into saturation at high gain and thus reduce the sensitivity. Programmable capacitors are therefore implemented on the chip; they are connected in parallel to each sensor's capacitor and have a value from 1 fF up to approximately 64 fF in steps of 1 fF. During calibration in a neutral atmosphere, the capacitance difference of each differential sensor is measured, the results are stored, and the total capacitance difference for each sensor is brought close to zero. The calibration procedure is especially important if more sensors are connected to one measurement channel because in this case, many spectral components are present in the measurement channel at the same time, and the intrinsic signal in the channel can be very big.

#### 3.3.4. Sensitivity and Noise Considerations

The charge amplifier (see amplifiers on Figure 12) is built of a high gain, single-ended, folded cascade low-noise operational amplifier with feedback impedance formed by parallel connection of *C_f_* and *R_f_*. The time constant is selected in such a way that the *S/N* at the output is optimal. The resistance *R_f_* must be very high, *i.e.*, in a range of several tens of GΩ in order to implement low pole frequency at small capacitor *C_f_* [29]. One charge amplifier processes charges from two differential sensors that are driven by excitation signals (V_S1_*_p_*, V_S1_*_n_*) and (V_S2_*_p_*, V_S2_*_n_*) with different frequencies.

The decisive parameter regarding the detection sensitivity of a measurement system is the signal to noise ratio at the output of the charge amplifier (2) in 1 Hz bandwidth around the corresponding spectral line. The details of those calculations are given in Supplementary file. The *S/N* ratio is proportional to the excitation signal amplitude *V_Sx_* and the ratio δ*C_x_/C_f_* and inversely proportional to noise of the amplifier *V*_ndop_ and the parasitic capacitance gain ratio given by (1 + ΣC_VG_/*C_f_*). These considerations instruct us about the optimum characteristics of the sensor-electronic pair:

(2) $$\left(\frac{S}{N}\right)≅\frac{V_{\text{Cho}}}{\sqrt{2}\cdot V_{\text{ndCho}}}=\frac{V_{Sx}\frac{\delta C_{x}}{\sqrt{2}C_{f}}}{\sqrt{3}\left[V_{\text{ndop}}\left(1+\frac{\sum C_{\text{VG}}}{C_{f}}\right)\right]}$$

The minimum detectable capacitance difference of sensor x can be calculated using Equation (3), taking into consideration that the signal power must be at least three times as large as the noise power in the specified band. The charge amplifier is designed with the following parameters:*V*_ndop_ (ω_x_) ≤ 7.5·10^−9^ V·*S*^1/2^, *V_Sx_* = 5 V, *C_f_* = 2 pF and Σ*C*_VG_ = 2 pF. The reduction of the *S/N* ratio caused by analogue and digital mixers is negligible, because each mixing contributes approximately $\sqrt{2}$, if designed properly [30,31]:

(3) $$\delta C≅3\sqrt{2}\frac{V_{\text{ndop}}}{V_{Sx}}\left(C_{f}+\sum C_{\text{VG}}\right)≅4.5\cdot 10^{-20}\;\left[\frac{F}{\sqrt{\text{Hz}}}\right]$$

For a comb capacitive sensor with the separation between two comb fingers of *d*_0_ ≅ 1 μm, and the initial capacitance of approximately *C*_0_ = 0.5pF, it is possible to estimate the minimum detectable distance change in plates δ_m_ between two fingers due to the adsorption according to Equation (4) and it is approximately 80 fm that is well below the thickness of one layer of adsorbed target molecules estimated to 0.5 nm [32] for the TNT. This fact can be used to measure the density of target molecules in the atmosphere around the sensor. The fact that δ_m_ is much smaller than the size of the target molecule makes the measurements possible even if the comb capacitor surface is not covered completely and the density of target molecules is smaller than the number of target molecules at vapour pressure:

(4) $$\delta_{m}≅\frac{d_{0}\cdot \delta C}{\left(C_{0}+\delta C\right)}≅8\cdot 10^{-14}\;m$$

#### 3.3.5. System Level Verification

To verify the concept, sensitivity, and functionality, the sensors and the whole electronic measurement system were modelled on a high hierarchical level using Matlab/Simulink; the most important non-ideal effects of the Integrated Circuit (IC) analogue measurement path were taken into consideration [33] as well as a bit-true model of the complete Digital Signal Processing (DSP) [34]. The system level simulation results are very close to the estimates, to the circuit simulation results, and to the measurements of the real circuit. The detailed results of the system level simulations are presented in the Supplementary information.

### 3.4. Response of a CE Demonstrator to TNT Vapour

**Figure 13.**
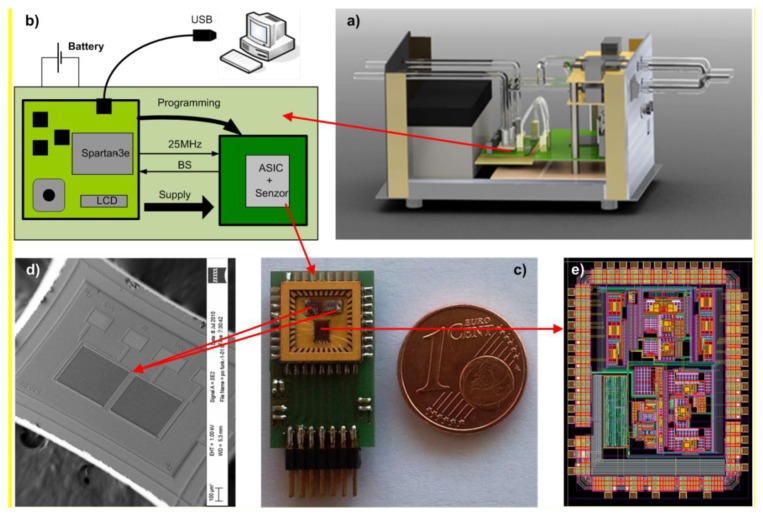
The demonstrator: (**a**) The complete demonstrator; (**b**) Complete electronics; (**c**) System in Package (SiP) with low-noise ASIC in 0.25 μm BDC technology and two differently modified sensors; (**d**) One differential sensor, and (**e**) Layout of the ASIC.

Figure 13a shows our demonstrator, which is built of the following modules:

(a) Complete demonstrator with all electronics, battery, piezoelectric pumps, and tubing
(b) The Printed Circuit Board (PCB) with ASIC, FPGA and all electronic components and the sensors
(c) Small PCB with some external passive components, System in Package (SiP) built of the ASIC and the sensors
(d) SEM micrograph of one differential sensor
(e) Micrograph of the ASIC in 0.25 μm BCD technology

The low-noise analogue front-end electronics of one measurement channel is integrated into the ASIC together with the excitation signal generators for four sensors. It performs the low noise analog signal processing of a composite signal from four sensors and a high resolution analog to digital conversion. Further signal processing is executed in the DSP, which is currently implemented on the FPGA. For a comparison of physical sizes, the one Euro cent coin is shown in Figure 13c. In reality, the package is covered and the gas with target molecules is delivered via Teflon tubes and exhausted through anothermtube.

### 3.5. Response of the Electronic Detection System to TNT and RDX Vapours

**Figure 14.**
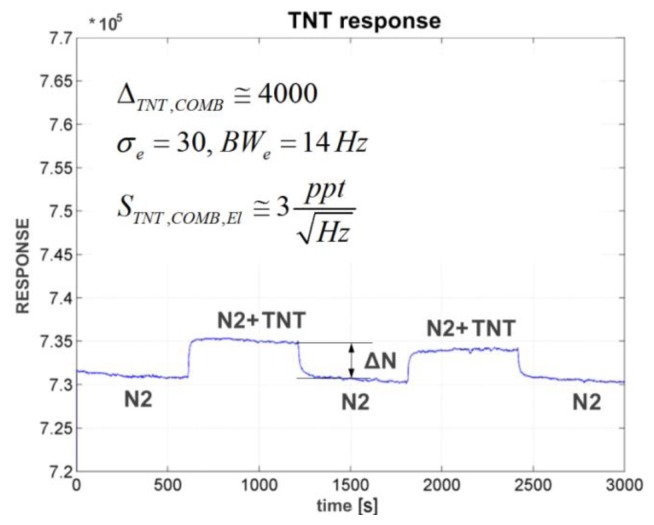
Measured response of a CE (Comb capacitive sensor with Electronic detection) surface-functionalized with APS molecules, to the gas, switched between pure N_2_ and N_2_ contaminated with 50% vapour pressure of TNT at room temperature. The normalized sensitivity of the sensor and the measurement system to the vapour of TNT is approximately 3 × 10^−12^. The RESPONSE is dimensionless and represents the number at the output of the DSP that is proportional to the relative change of the capacitance δ*C_x_/C_f_*.

The measurements were performed in the controlled laboratory environment using a gas generator as described in Section 4.1, as well as in a real environment. The sampling gas was pumped to the sensor using miniature piezoelectric pumps with a flow rate of 15 mL/min. For laboratory experiments, the gas input was switched between dry N_2_ gas and the N_2_ contaminated with target molecules in equal proportion. At room temperature, the vapour pressure of the TNT is 6 × 10^−4^ Pa, which means that the density of target TNT molecules relative to the N_2_ molecules is at the vapour pressure in a range of *X_t_*_arg_ = 10^−9^ [32]. Figure 14 shows the response of one channel to the gas switched between pure N_2_ and N_2_ contaminated with TNT in equal proportions. From the difference between the two readings, Δ*N*_TNT,CE_ = 4000, the standard deviation σ_CE_ = 30 and the bandwidth *BW*_CE_ = 14 Hz and taking into account the fact that the signal must be at least three times as large as the background noise (CE stands for **C**apacitive sensing with **E**lectronic detection), one can estimate the normalized sensitivity of the sensor and measurement system for TNT target molecules using Equation (5):

(5) $$S_{\text{TNT},\text{CE}}≅\frac{0.5\cdot X_{t\arg}\cdot \sigma_{\text{CE}}\cdot 3}{\Delta N_{\text{TNT},\text{CE}}\sqrt{BW_{\text{CE}}}}=\frac{3\cdot 10^{-12}}{\sqrt{\text{Hz}}}$$

The digital response at the output of the DSP *N*_out_ is dimensionless and can be estimated using Equation (6), which is derived from the equations given in the Supplementary Information:

(6) $$N_{\text{out}}≅\frac{V_{Sx}}{V_{\text{ref}}}\cdot \frac{\delta C_{x}}{C_{f}}\cdot G_{\text{A}}\cdot G_{\text{D}}$$

Here, *V_Sx_* is the excitation signal amplitude, *V*_ref_ is the reference voltage of the ADC, *G*_A_ is the gain of complete analogue measurement path including the gain of the ADC and the *G*_D_ is the gain of the DSP part of the signal processing. The offset on the y-axis in Figure 14 is proportional to the remaining intrinsic difference between the capacitors *C_P_* and *C_n_* (ΔC = C*_p_* − *C_n_*) of one differential sensor after the calibration, while the change due to the adsorbed molecules is proportional to Δ*N*_TNT,COMB_. Rather long measurement times are a consequence of a slow flow of the sampling gas through the piezoelectric diaphragm pumps used (gas flow ∼15 mL/min), and not because of any electronic measurement delay and averaging. Similar laboratory measurements were performed for other target molecules.

**Figure 15.**
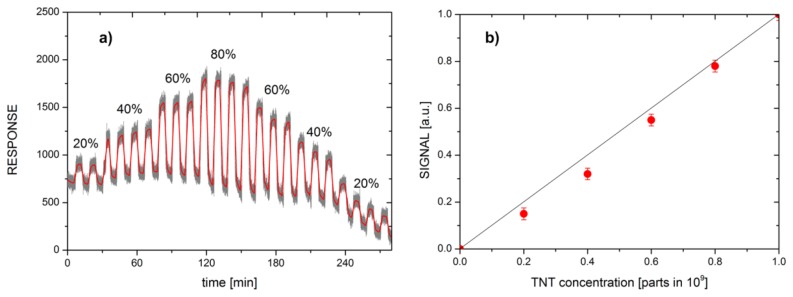
Response of a CE measurement system using comb sensors, surface-functionalized with APS (CE-APS), to various concentrations of TNT vapours in the carrier gas N_2_. (**a**) Time-dependence of the CE-APS response to the gas, switched between pure N_2_ and N_2_ with added TNT vapours of different concentrations. The concentration of TNT was first increased in steps of 20% and then decreased back. Black symbols represent raw data, red symbols are 2000 point averaged data. (**b**) Signal of the CE-APS measurement system as a function of peak concentration of TNT in N_2_.

Figure 15 shows the concentration dependence of the response of CE measurement system, using the comb sensor that was surface-functionalized with APS molecules. Panel (a) shows the time dependence of CE-APS response to the gas, switched between pure N_2_ and N_2_ with added TNT molecules in controlled concentration. During the measurement, the concentration of TNT molecules was increased in steps of 20% of the vapour pressure of TNT. The amplitude of the response of CE-APS measurement system to various TNT concentrations is shown in Figure 15b.

## 4. Vapour Generator and Calibration

### 4.1. Vapour Generator

**Figure 16.**
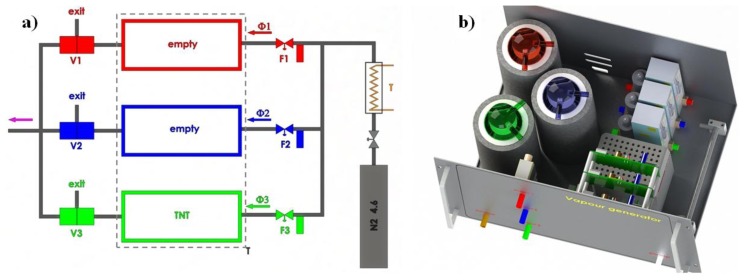
(**a**) Schematic diagram of the vapour generator. N_2_ gas from a storage tank is thermally stabilized (T) and divided into three parallel flow lines (Φ1, Φ2, Φ3), each with electronic flow regulator (F1, F2, F3). The switching between the pure carrier gas and gas with the known concentration of the explosive's vapour, while keeping the total mass flow constant, is done by mixing the gas from an empty glass cylinder (Φ1) with either pure gas (Φ2) or with saturated gas (Φ3). The mixing is controlled by three valves V1, V2 and V3. (**b**) A model of the actual vapor generator used in the experiments. Red, blue, and green colors represent individual flow lines.

**Figure 17.**
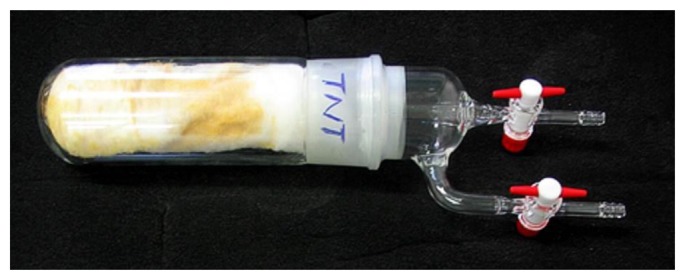
Photograph of a glass cylinder with two Teflon valves on the top. approximately 5 mg of 2× recrystallized TNT was deposited onto glass wool.

The development and testing of explosive vapour trace detector requires a reliable gas generator, capable of producing known, controllable, and very low concentrations of explosive vapours. We have developed and tested a computer-controlled vapour generator, which allows for long-term and reliable generation of TNT and other vapours with concentrations in the range from 0.05 to 1 vapour pressure of the selected element. The schematic diagram is presented in Figure 16a and a model of a constructed vapour generator in Figure 16b. A N_2_ carrier gas from a storage tank passes through a regulated resistive heater, which heats the flowing gas to a pre-set temperature with an accuracy of ± 50 mK. The flow of this thermally stabilized gas is divided into three parallel lines, where the gas flow through each line is regulated by an electronic flow controller (Aalborg GFC17A, Orangeburg, NY, USA). Each flow line is connected to one of the three equivalent glass vessels, which are thermally stabilized to any pre-set temperature in the range 25 °C to 65 °C with an accuracy of ±50 mK. One of the cylinders contains a known mass of the explosive under consideration, which was deposited on glass wool (Figure 17), while the other two cylinders were empty. The switching and precise control of the concentration levels of the explosive's vapour in the output gas is achieved by mixing the explosive-saturated gas with the pure carrier gas.

At all times, the total mass flow Φ is always kept constant by matching the flows Φ1 and Φ3. The electronic 3-way valves V1, V2, and V3 control the output of the vapour generator: the mixing of a pure Φ2 and Φ1 gas results in a reference gas flow without any traces of explosive vapours, while mixing of a pure Φ2 gas with saturated Φ3 explosive vapours delivers gas with known concentrations of explosive. Additionally, the concentration of the vapours can be controlled by varying the temperature of the gas and the glass cylinders, thus varying the vapour pressure of the explosive.

Great care was taken to avoid any contamination of the system. All the components were made out of stainless-steel, Teflon, or glass and thoroughly cleaned and flushed with carrier gas for a longer time prior to any usage in the detection experiments. The temperature stability of the output gas with a known concentration of the explosive's vapour was found to be better than ±1 mK, and the concentration was stable over weeks of constant operation.

**Figure 18.**
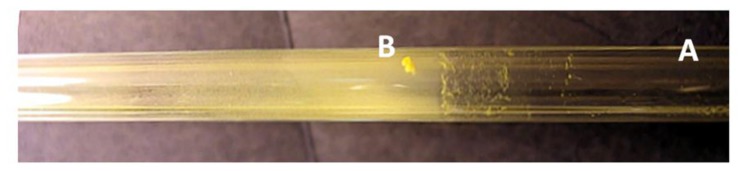
Photograph of a glass tube, used for cold trapping of TNT molecules from the gas phase. The right side (A) of the glass tube was close to room temperature, while the left side (B) was immersed in liquid nitrogen during purging with 1000 L of N_2_ carrier gas with TNT molecules.

**Figure 19.**
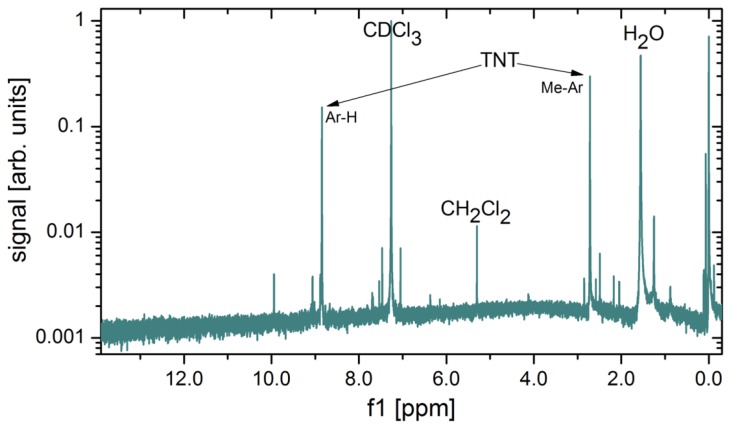
NMR spectra of the material collected from 1000 L of gas mixture using a cold trap. The flow through a glass tube immersed in liquid nitrogen was set to 100 mL/min (as during normal operation of vapor generator). A peak of solvent deuterated chloroform (CDCl_3_), a water peak (H_2_O), a CH_2_Cl_2_ peak, and TNT peaks (Me–Ar and Ar–H) can clearly be seen, all other impurities are present only in traces. CH_2_Cl_2_ is a solvent, which was used for deposition of TNT onto glass wool.

Several methods for calibration of the concentration of explosive vapours in the output gas were tested. It turns out that the most precise and reliable method of measuring the very low concentrations of explosive vapours from the generator's output is also the simplest one. The output of the vapour generator is connected to a glass tube, and immersed in the liquid nitrogen at 77 K. As the gas flows through the cold part, the solid and liquid fractions present in the carrier gas gradually condense at the tube's surface. At a flow rate of 100 mL/min, one can clearly observe solid condensate and water droplets on the glass surface after several days of gas flow. It takes typically 150 h to collect measurable quantities of solidified condensate (Figure 18), which is then weighed, while its chemical composition is checked with nuclear magnetic resonance (NMR) spectroscopy (Oxford Instruments, Dallas, TX, USA). A NMR spectrum of a material condensed from 1000 L of gas mixture is shown in Figure 19. The strongest peak is attributed to the deuterated chloroform (CDCl_3_), which is a solvent used in our NMR analysis. Water (H_2_O), which is present in the nitrogen gas as an impurity, is also found in large quantities. Two TNT peaks (from Ar–H and Me–Ar) are clearly seen. A peak at around 5.2 ppm is attributed to CH_2_Cl_2_, a solvent, which was used to dissolve and deposit TNT onto the glass wool. Although CH_2_Cl_2_ is very volatile it was still found in traces in gas mixture after several weeks of purging with clean N_2_.

## 5. Sensitivity Comparison

The sensitivity comparison of vapour trace-detection of explosives based on CMO (chemo-mechanical sensing with optical detection) and CE (capacitive censing with electronic detection) is based on Equation (5) by normalizing both responses to approximately the same conditions; for some parameters this is possible, for the others it is not, because of a different mechanism of operation. To calculate the figure of merit (FOM), one needs to first calculate the sensitivity under approximately the same conditions regardless of the principle, volume, power consumption, *etc.*, but at the same density of target molecules, the same pressure, the same temperature, and the same measurement bandwidth. The same vapour pressure is assured by using the same vapour generator as explained in Section 4.1. The bandwidth of each measurement is normalized to 1 Hz; the temperature is kept constant during the measurements (constant temperature is important for chemo-mechanical sensing and much less for capacitive sensing). The comparison of two sensitivities is in favour of capacitive sensing with electronic detection since $S_{\text{TNT},\text{CE}}≅3\cdot 10^{-12}/\sqrt{\text{Hz}}$ is smaller than $S_{\text{TNT},\text{CMO}}≅300\cdot 10^{-12}/\sqrt{\text{Hz}}$. This conclusion is valid at room temperature and at normal pressure. For extreme conditions where AFM is usually used (very good vacuum and extremely low temperature) the chemo-mechanical sensing with optical detection would most likely outperform the capacitive sensor with the electronic detection system by several orders of magnitude.

Since the application requires operation at room temperature and normal pressure, and with as simple handling as possible, the CE system outperforms the CMO system, not only in the sensitivity, but also in functionality, temperature independence, volume, power consumption, construction simplicity and the stability of its operation, and potential price. The results of a comparison based on our comparative study are presented in Table 1.

## 6. Conclusions

The results of our systematic, comparative study clearly suggest that at this stage of technology, capacitive sensing with electronic detection (CE) of vapour traces of explosives in the atmosphere is far superior to the existing solutions based on chemo-mechanical (CMO) sensing using MEMS with an optical detection. The advantage of CE is not only in nearly two-orders of magnitude higher sensitivity to vapours, but also in a much better temperature stability of sensors and detection equipment, robustness to mechanical (acoustic) noise from the environment, and much smaller volume and power consumption. Moreover, we expect that by further integration of comb sensors and by decreasing the spacing between the electrodes of comb sensors, the sensitivity of the CE method could be increased by an additional two orders of magnitude. This means that detection of 1 molecule of TNT in 10^+14^ molecules of a carrier N_2_ could be achieved in the near future.

## Figures and Tables

**Table 1.** Comparison of important characteristics of CMO and CE systems (CMO stands for Chemo Mechanical sensing with Optical detection and CE stands for comb Capacitive sensing with Electronic detection).

**Parameter**	**CMO**	**CE**
Sensitivity	300 × 10^−12^	3 × 10^−12^
volume	cm^3^	mm^3^
Temperature stability	poor	very good
Sensitivity to vibrations	very sensitive	insensitive
Power consumption	big	15 mA
functionality	complicated	simple
Sensitivity in a vacuum and at low temperature	very good	moderate
